# Conditional flux balance analysis toolbox for python: application to research metabolism in cyclic environments

**DOI:** 10.1093/bioadv/vbae174

**Published:** 2024-11-13

**Authors:** Timothy Páez-Watson, Ricardo Hernández Medina, Loek Vellekoop, Mark C M van Loosdrecht, S Aljoscha Wahl

**Affiliations:** Department of Biotechnology, Delft University of Technology, Delft 2629 HZ, The Netherlands; Department of Biotechnology, Delft University of Technology, Delft 2629 HZ, The Netherlands; Department of Biotechnology, Delft University of Technology, Delft 2629 HZ, The Netherlands; Department of Biotechnology, Delft University of Technology, Delft 2629 HZ, The Netherlands; Lehrstuhl für Bioverfahrenstechnik, Friedrich-Alexander University Erlangen-Nürnberg, Erlangen 91052, Germany

## Abstract

**Summary:**

We present py_cFBA, a Python-based toolbox for conditional flux balance analysis (cFBA). Our toolbox allows for an easy implementation of cFBA models using a well-documented and modular approach and supports the generation of Systems Biology Markup Language models. The toolbox is designed to be user-friendly, versatile, and freely available to non-commercial users, serving as a valuable resource for researchers predicting metabolic behaviour with resource allocation in dynamic-cyclic environments.

**Availability and implementation:**

Extensive documentation, installation steps, tutorials, and examples are available at https://tp-watson-python-cfba.readthedocs.io/en/. The py_cFBA python package is available at https://pypi.org/project/py-cfba/.

## 1 Introduction

Optimal resource allocation is a widespread theory used to study evolutionary trade-offs—inherent in metabolic processes (Molenaar et al. 2009, Flamholz et al. 2013, Vázquez-Laslop and Mankin 2014, Elsemman et al. 2022). The prevailing literature predominantly focuses on microorganisms thriving under stationary conditions (Molenaar et al. 2009, Flamholz et al. 2013, Vázquez-Laslop and Mankin 2014, Elsemman et al. 2022). While such conditions lend themselves to laboratory validation, in reality, microbial habitats in nature and most environmental biotechnology applications are far from static. Over evolutionary timescales, microorganisms have evolved diverse metabolic strategies to face a diverse array of dynamic environmental fluctuations (Bajic and Sanchez 2020, Caetano et al. 2021). These fluctuating environments and the metabolic strategies of organisms living therein can be studied with computational models. Modelling is key to establish fundamental principles governing evolutionary fitness.

Rügen et al. (2015) introduced a mathematical framework named conditional flux balance analysis (cFBA), designed to predict optimal resource allocation dynamics under fluctuating conditions. This framework has been applied to cyanobacteria (Reimers et al. 2017) and polyphosphate accumulating organisms (Páez-Watson et al. 2023). In both cases, temporal synthesis of storage polymers (e.g. glycogen, polyphosphate, and polyhydroxyalkanoates) resulted as an emergent property of resource optimization in dynamic-cyclic scenarios. Nevertheless, reports on dynamic conditions remain sparse, primarily confined to these exemplar cases.

The cFBA method integrates stoichiometric modelling, dynamic Flux Balance Analysis (dFBA) with a final optimization through the whole simulation time, and resource allocation to study metabolic dynamics within cyclic environments. As such, it serves as a potent predictive tool for unveiling optimal metabolic strategies in ecosystems such as diurnal cycles, feast–famine dynamics, and aerobic–anaerobic transitions. Given the prevalence of such environmental conditions in nature, the field of microbial ecology could benefit from the application of cFBA. To date, there is no cFBA tool based on open-source platforms. The current implementation relies on adoption of complex metabolic models in MATLAB (Rügen et al. 2015, Reimers et al. 2017) with little documentation or simple examples for its application. Here, we present an easy-to-use Python toolbox for the application of cFBA (see Table 1 for a comparison of py_cFBA with other published models). This toolbox allows users to explore the boundaries of metabolic behaviour given a stoichiometric model, enzyme capacities, and a set of environmental conditions.

**Table 1.** A comparison of cFBA with similar methods to research dynamic metabolism.

Feature	RBApy (Bulović et al. 2019)	Cycle Sync	cFBA	py_cFBA (this research)
Method	Resource Balance Analysis (Goelzer et al. 2015)	Cycle Sync (Sarkar et al. 2019)	Conditional FBA (Rügen et al. 2015)	Conditional FBA (Rügen et al. 2015)
Language	Python	Python	MATLAB^a^	Python
Toolbox for user implementation	+	–	–	+
Comprehensive documentation, tutorials and examples	+	–	–	+
Temporal flux analysis	+	+	+	+
Enzyme constraints	+	–	+	+
Study of organisms in cyclic environments	–	+	+	+
Capture temporal use of storage polymers	–	+	+	+

a Requires a license for its use.

## 2 Toolkit usage and user input

The cFBA toolkit is accessible as a Python package https://pypi.org/project/py-cfba/. It comprises a suite of functions enabling the construction of the cFBA model architecture and subsequent simulations. The required user input varies depending on the desired complexity of the analysis. For instance, to model dynamic cycling without any catalytic information, a stoichiometric matrix suffices. However, if enzyme capacities are to be included, numerical constants describing the relationships between reactions and their respective catalytic efficiencies (k_cat_) are needed. The first step in this toolkit’s pipeline is to generate a basic cFBA model structure, which is encoded into Systems Biology Markup Language (SBML). Subsequently, the SBML model is parsed into a linear programming problem. Detailed instructions on model generation and SBML file creation can be found at https://tp-watson-python-cfba.readthedocs.io/en/.

## 3 Methods and implementation

### 3.1 Minimal set of constraints: unlimited catalytic activity

The underlying metabolic model in cFBA is represented by a stoichiometric matrix (S), which represents the interplay of metabolites and reactions in a metabolic network. From the participating species (metabolites enzymes and biomass components), a subset is expressed as imbalanced (*M*). These species—typically enzymes, ribosomes, membranes, storage polymers, and substrates—exhibit explicit concentration changes over time, which are explicitly modelled as done with dFBA (Mahadevan et al. 2002). Conversely, the remainder of metabolites ($\bar{M}$) are presumed to remain in quasi-steady state, because their turnover rate is significantly faster than that of imbalanced species (Rügen et al. 2015, Waldherr et al. 2015, Reimers et al. 2017). Users can simulate dynamic environmental changes (such as variations in light, substrate and oxygen) by constraining reactions with upper and lower bounds.

Each cFBA simulation is normalized to an initial amount of biomass [typically 1 gram dry weight (g_DW_)]. Biomass is not modelled as an independent metabolite with its corresponding biosynthesis reaction, but rather defined as the weighed sum of all components in *M* at each time point (all imbalanced metabolites). To normalize the initial time point of the simulation to 1 g_DW_, (1) is employed (Rügen et al. 2015).

(1) $${w^{T}M}^{t=0}=1$$

Here, *w^T^* represents the transpose of a matrix containing the molecular weights of each imbalanced metabolite in *M*. The cyclic behaviour of cFBA is achieved by enforcing identical relative amounts of imbalanced metabolites at both the beginning and end of the simulation (2) (Rügen et al. 2015).

(2) $$M^{t_{\mathrm{end}}}=\mu \;M^{t=0}$$

µ represents the balanced growth of the system. These constraints represent a quadratic programming problem, which becomes linear for each value of µ. The objective of the cFBA model is to achieve the highest multiplication factor (µ) using a binary search algorithm. Numerically-stable solvers with high numeric precision, such as Gurobi, are recommended since complex models may lead to ill-conditioned problems (Reimers et al. 2017). The implementation of this method uses the OPTLANG library in Python and the solvers supported and their limitations have been described (Sonnenschein et al. 2018).

### 3.2 Cellular limits and requirements on metabolites: quotas

By default, the synthesis of imbalanced metabolites is not enforced [apart from maintaining the relation in (1)]. Minimal cellular requirements can be enforced by setting quota definitions (minimal concentration constraints). For instance, Rügen et al. (2015) employed quota compounds to establish minimal thresholds for inorganic ions, cell wall constituents, lipids, DNA, and non-catalytic proteins, relative to biomass. Expanding upon the quota definitions utilized by Rügen et al. (2015) and Reimers et al. (2017), our toolkit enables users to define exact, minimum, and maximum quota constraints at any time point during the simulation. This facilitates the capture of dynamic behaviours in simulated environmental conditions.

### 3.3 Coupling metabolism to protein allocation: enzyme activity based on enzyme amounts

Imbalanced metabolites can also act as catalysts of specific reactions. The relation between the metabolite and the reaction it catalyses is indicated in the capacity matrices [A and B in (3)], which denote the associations between catalysed reactions and the k_cat_ values of each catalysed reaction (Rügen et al. 2015).

(3) $${A_{\mathrm{cap}}}^{t}{v_{r}}^{t}\leq B_{\mathrm{cap}}\cdot M^{t}$$

(4) $${v_{r}}^{t}\leq {M_{e}}^{t}\cdot k_{{\mathrm{cat}}_{e}}^{r}$$

Equation (3) sets a reaction *r* catalysed by an imbalanced metabolite *M_e_* to be constrained by its upper limit following the relation in (4). It is noteworthy that this defines an upper boundary to the reaction, not an exact value. Additionally, if a reversible reaction is catalysed by an imbalanced specie, both directions of the reaction must be accounted for in the S matrix.

Standard genome-reconstructed metabolic models typically include storage polymers such as glycogen as part of biomass components. Following the approach of Ofaim et al. (2021), we allow for the explicit separation of storage metabolites from that of biomass (also referred to as *lean* biomass). This allows the independent accumulation and utilization of said polymers in various simulations irrespective of biomass composition [independent from (2)].

**Figure 1.**
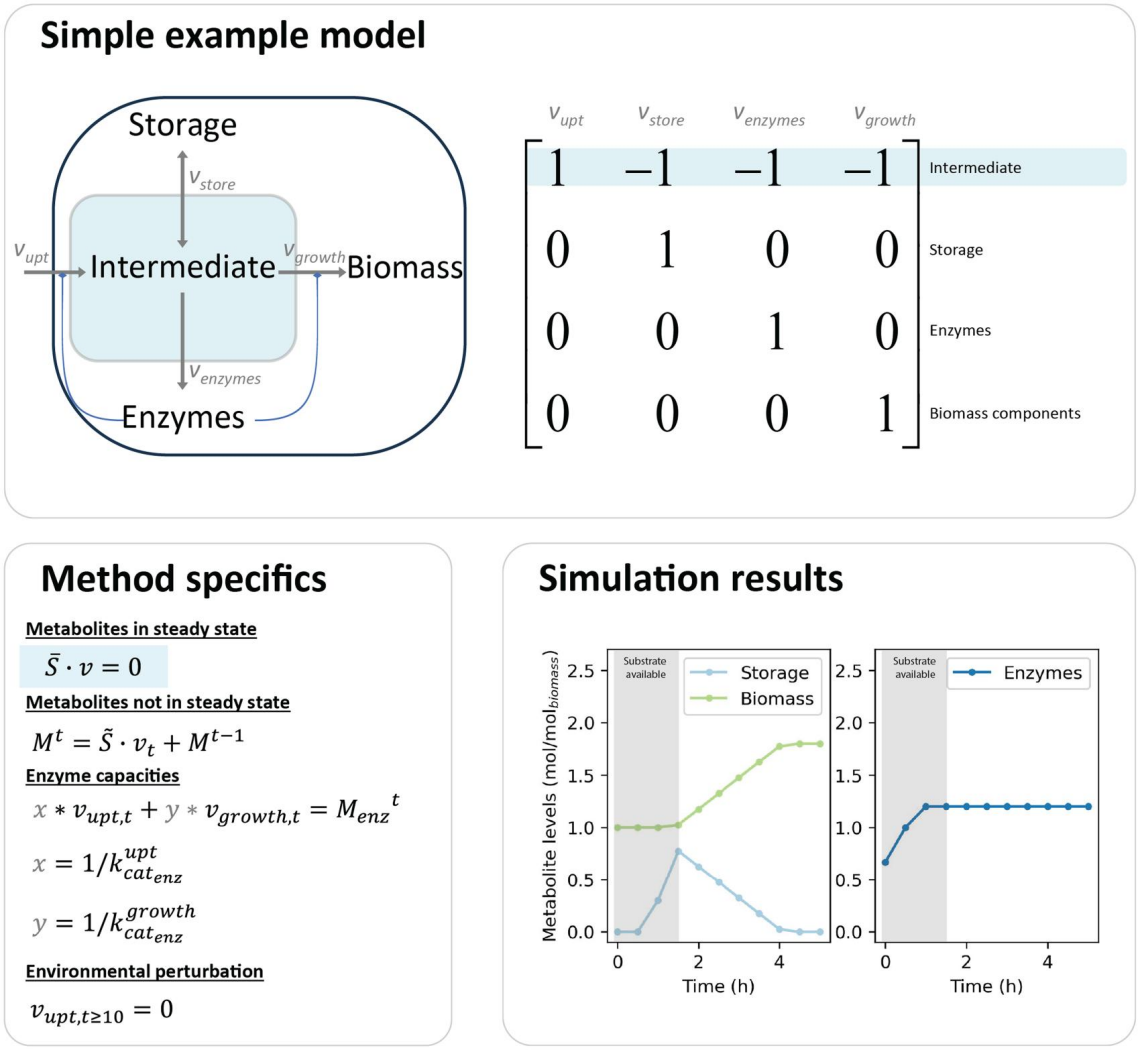
Basic simulation of a toy-model using the cFBA Python Toolbox. Inputs required for this model are a stoichiometric matrix, definition of balanced and imbalanced metabolites, and enzyme capacities. The simulation includes an active feed during the first 10 units of time after which there is no longer substrate simulating a feast–famine condition. Specifics and step-by-step implementation of this model in the cFBA python toolbox are available at https://tp-watson-python-cfba.readthedocs.io/en.

An illustrative example of cFBA implementation for a toy model of a minimal cell is presented in Fig. 1. The system comprises one balanced metabolite (named ‘intermediate’) and three imbalanced species: storage, enzymes, and biomass. The reactions for substrate uptake and biomass synthesis are catalysed by the species ‘enzymes’ each with a distinct k_cat_ value. The simulation incorporates a dynamic component wherein substrate is only available until 2 h in the simulation. No quota compounds are defined, and the metabolite ‘*biomass*’ solely contributes to *w^T^.* The cFBA simulation results in an early use of resources (substrate) into enzyme biosynthesis to reach the maximum catalytic capacity at the third time-point (1 h). After this point, the system produces biomass at a balanced rate (optimizing enzyme usage), making temporal use of storage to allow this steady rate of biomass synthesis. Variations and step-by-step examples of this model implementation are available at https://tp-watson-python-cfba.readthedocs.io/en.

## 4 Conclusion

The Python cFBA toolkit facilitates the study of metabolic dynamics in cyclic environments. We included clear documentation and examples for a fast familiarization to resource allocation strategies in dynamic conditions. Two considerations are of note: numerical challenges may require specialized solvers, and further developments are needed to address complex biological systems such as non-optimal balanced growth strategies or microbial communities. Notwithstanding, the toolkit represents a significant advancement in systems biology, offering researchers a powerful tool to explore metabolic behaviour in dynamic-cyclic environments.

## Data Availability

Extensive documentation, installation steps, tutorials and examples are available at https://tp-watson-python-cfba.readthedocs.io/en/. The py_cFBA python package is available at https://pypi.org/project/py-cfba/.
